# Using deep learning to safely exclude lesions with only ultrafast breast MRI to shorten acquisition and reading time

**DOI:** 10.1007/s00330-022-08863-8

**Published:** 2022-05-26

**Authors:** Xueping Jing, Mirjam Wielema, Ludo J. Cornelissen, Margo van Gent, Willie M. Iwema, Sunyi Zheng, Paul E. Sijens, Matthijs Oudkerk, Monique D. Dorrius, Peter M.A. van Ooijen

**Affiliations:** 1 Department of Radiation Oncology, and Data Science Center in Health (DASH), Machine Learning Lab, University Medical Center Groningen, University of Groningen, Hanzeplein 1, 9713 GZ Groningen, The Netherlands; 2 Department of Radiology, University Medical Center Groningen, University of Groningen, Hanzeplein 1, 9713 GZ Groningen, The Netherlands; 3 Department of Radiation Oncology, University Medical Center Groningen, University of Groningen, Hanzeplein 1, 9713 GZ Groningen, The Netherlands; 4 Faculty of Medical Sciences, University of Groningen, Antonius Deusinglaan 1, 9713 AV Groningen, The Netherlands; 5 Faculty of Medical Sciences, University of Groningen and Institute of Diagnostic Accuracy, Wiersmastraat 5, 9713 GH Groningen, The Netherlands

**Keywords:** Breast neoplasms, Mass screening, Magnetic resonance imaging, Deep learning

## Abstract

**Objectives:**

To investigate the feasibility of automatically identifying normal scans in ultrafast breast MRI with artificial intelligence (AI) to increase efficiency and reduce workload.

**Methods:**

In this retrospective analysis, 837 breast MRI examinations performed on 438 women from April 2016 to October 2019 were included. The left and right breasts in each examination were labelled normal (without suspicious lesions) or abnormal (with suspicious lesions) based on final interpretation. Maximum intensity projection (MIP) images of each breast were then used to train a deep learning model. A high sensitivity threshold was calculated based on the detection trade - off (DET) curve on the validation set. The performance of the model was evaluated by receiver operating characteristic analysis of the independent test set. The sensitivity, specificity, positive predictive value (PPV), and negative predictive value (NPV) with the high sensitivity threshold were calculated.

**Results:**

The independent test set consisted of 178 examinations of 149 patients (mean age, 44 years ± 14 [standard deviation]). The trained model achieved an AUC of 0.81 (95% CI: 0.75–0.88) on the independent test set. Applying a threshold of 0.25 yielded a sensitivity of 98% (95% CI: 90%; 100%), an NPV of 98% (95% CI: 89%; 100%), a workload reduction of 15.7%, and a scan time reduction of 16.6%.

**Conclusion:**

This deep learning model has a high potential to help identify normal scans in ultrafast breast MRI and thereby reduce radiologists’ workload and scan time.

**Key Points:**

*• Deep learning in TWIST may eliminate the necessity of additional sequences for identifying normal breasts during MRI screening.*

*• Workload and scanning time reductions of 15.7% and 16.6%, respectively, could be achieved with the cost of 1 (1 of 55) false negative prediction.*

**Supplementary Information:**

The online version contains supplementary material available at 10.1007/s00330-022-08863-8.

## Introduction

Dynamic contrast-enhanced MRI (DCE-MRI) of the breast has been widely used as a supplementary screening tool for breast cancer. Breast MRI can not only detect more breast cancer cases than mammography but also detect cancers at an earlier stage [1]. Especially for women with extremely dense breasts, screening with supplemental MRI has the potential to reduce interval cancers [2]. These advantages have led to a renewed interest in using breast MRI to screen a larger population [3]. However, cost-effectiveness is still the most substantial obstacle for the wider application of this sensitive modality [4].

The most promising approaches to reducing the cost of breast MRI are to improve the throughput of the MRI scanner by shortening the acquisition time [5–8] and reducing radiologists’ workload by shortening the interpretation time [9]. Current diagnostic breast MRI protocols require up to 20 min. Several abbreviated protocols have been proposed to replace the standard protocol for screening [10, 11]. A recent multicenter, multireader study [12] found that time-resolved angiography with stochastic trajectories (TWIST) [13] alone can achieve a comparable sensitivity (84% vs. 86%) and higher specificity (82% vs. 76%) than the full diagnostic protocol when interpreted by radiologists. This TWIST-alone protocol, requiring less than 2 min of magnet time, can thus minimize the time needed for the scanning process.

Image interpretation is another bottleneck in breast cancer screening with MRI. The average interpretation time in different studies varied from 25 to 178 s [11]. It is worth noting that the cancer rate in a screening study may be only 15.5 per 1000 [14], which suggests that radiologists spend most of their time reading normal scans without suspicious lesions. On the other hand, reading quality is also related to the total number of examinations and the position of the examination in the queue [15]. Short reading batches and risk-based reading queues may help further improve radiologists’ performance.

The combination of artificial intelligence (AI) and ultrafast MRI could help improve the efficiency of breast MRI screening by automatically excluding scans without lesions. Identifying suspicious lesions from numerous screening scans and prioritizing a scan according to risk could help reduce the workload and improve efficiency. In addition, an early stop strategy could also be applied to scans without suspicious lesions. Since malignant lesions are more likely to enhance rapidly at the early stage of DCE-MRI [16, 17], cancellation or adjustment of further sequences based on the output of ultrafast MRI could help reduce scanning time and thus improve the throughput. Moreover, based on the real-time analysis of the ultrafast sequences, additional scanning (e.g., T2, DWI) or even a full diagnostic protocol could still be performed if any abnormalities were detected.

We hypothesized that a deep learning model, with only TWIST sequences as input, might be able to identify normal MRI exams without human intervention. Integrating this deep learning system in the screening workflow could improve the throughput and reduce the radiologist’s workload. Therefore, the aim of this study was to develop and evaluate a deep learning model for automated abnormality prediction with only TWIST sequences as input.

## Materials and methods

The institutional review board approved the study and waived the requirement to obtain informed consent for our retrospective study, which used fully anonymized reports and MRI examinations.

### Study population

**Fig. 1:**
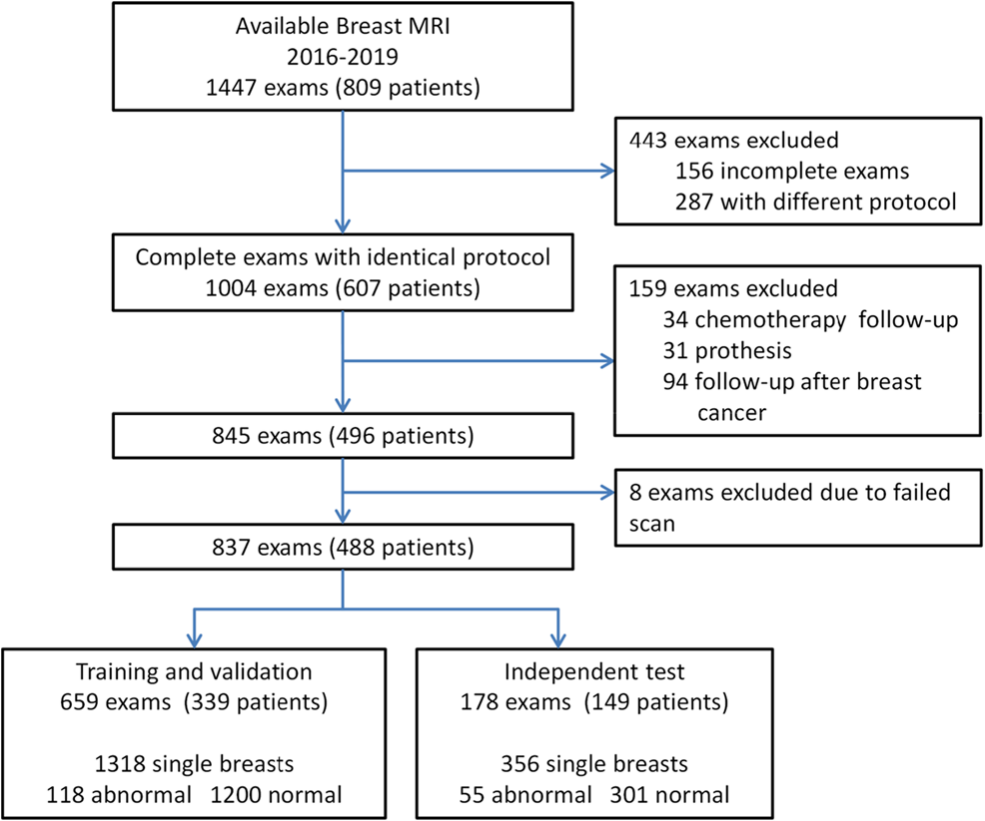
Flowchart of the data collection and selection procedure. BI-RADS, Breast Imaging Reporting and Data System

The initial population included 1447 breast MRI examinations from 809 consecutive patients who underwent breast MRI examinations between April 2016 and October 2019 at our institution. Of the 1447 examinations, the following MRI scans were excluded: 287 due to inconsistent protocols, 156 due to incomplete data, and 159 due to another indication for scanning (34 to measure response to chemotherapy, 94 for surgery follow-up, and 31 to evaluate prosthesis rupture). Furthermore, 8 examinations were excluded due to failed scans. The final dataset for deep learning model development and evaluation consisted of 837 examinations from 488 patients. Among the 837 examinations, 178 examinations from 149 patients were obtained after deep learning model development, and those data were used as an independent test set since they were not involved in the model development. The remaining 659 examinations from 339 patients were randomly divided into training and validation sets as follows: 494 examinations from 214 patients in the training set and 165 examinations from 125 patients in the validation set. It should be noted that the data were divided on the patient level; thus, there was no overlap in patients in the training and test sets. Figure 1 summarizes this process.

### MRI scanner and imaging technique

**Fig. 2:**
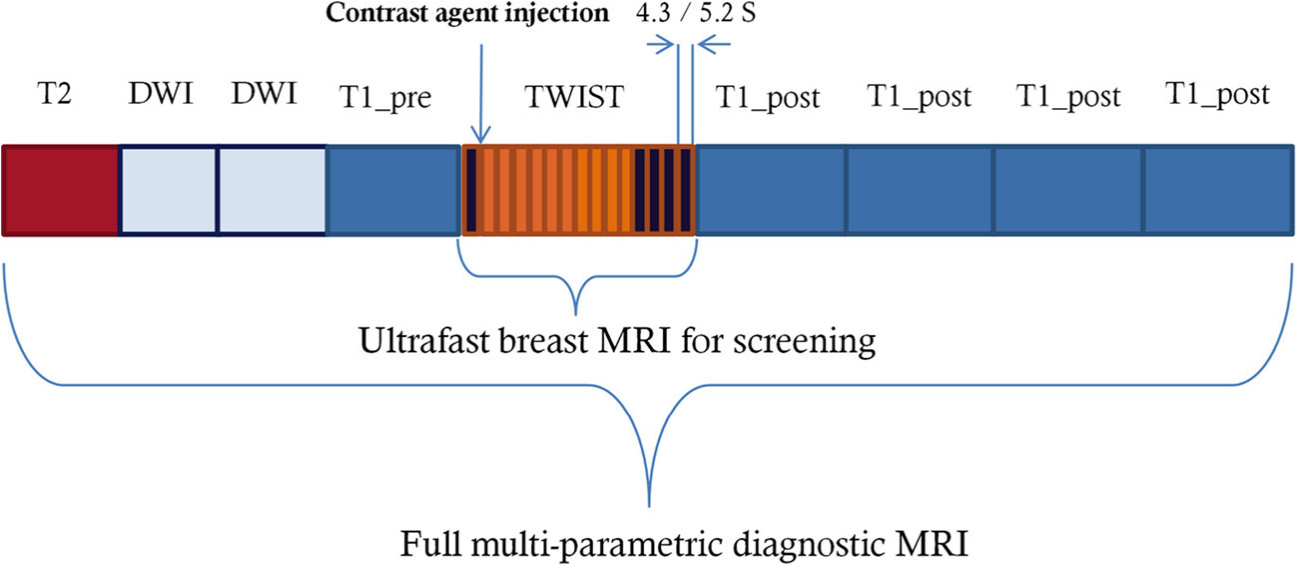
Schematic diagrams of the timing of dynamic contrast-enhanced protocols used in this study. The full diagnostic protocol consists of a fat-saturated T2-weighted sequence, 2 diffusion-weighted imaging sequences with *b*-values of 0 and 1000 s/mm^2^, 5 dynamic fat-saturated gradient echo T1-weighted sequences, and a time-resolved angiography with stochastic trajectories (TWIST) sequence

Examinations were performed with a full diagnostic protocol (Fig. 2) on either a 3.0-T or 1.5-T scanner (MAGNETOM Skyra or MAGNETOM Avanto^fit^, Siemens Healthineers) in the prone position. For 3.0-T and 1.5-T scanners, the full protocol requires 17.95 and 19.61 min, respectively, while the 15 TWIST acquisitions require 1.3 and 1.46 min. The acquisition parameters for ultrafast breast MRI are summarized in Table 1.

**Table 1:** Acquisition Parameters for ultrafast MRI

Parameter	1.5 T	3.0 T
TR/TE, ms	2.50/0.90	4.12/2.08
Flip angle (°)	20	20
Voxel size (mm^3^)	0.68 × 0.68 × 3.0	0.91 × 0.91 × 3.0
Temporal resolution (s)	5.2	4.3
FOV (mm)	350	350
Fat suppression	No	No

*TR* repetition time, *TE* echo time, *FOV* field of view

### Reference standard

Classification of the MRI examinations was based on the assessments and conclusions in the radiology reports, supplemented with pathology reports, biopsy, and ultrasound results. For each patient, the left and right breasts were evaluated independently. Breasts with one or more visible enhanced lesions were classified as abnormal, while breasts with unenhanced lesions or without suspicious lesions were classified as normal. Then, all the labels were further examined by a senior radiologist to ensure that they were consistent with the visibility in TWIST. Examples of classified breasts are shown in Electronic supplementary material Fig. S1.

### Development of the MIP-based deep learning system

**Fig. 3:**
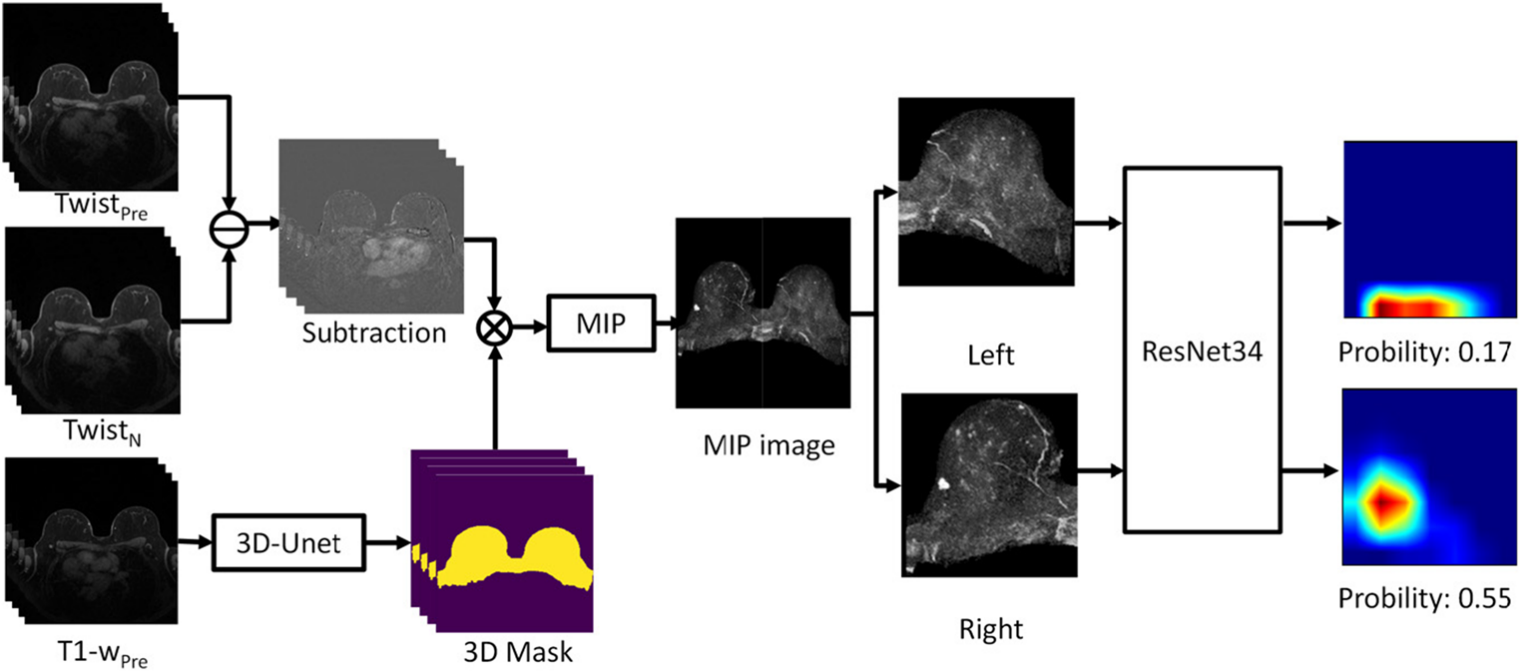
Schematic flowchart of the proposed breast DCE-MRI classification system. *TWIST*_*Pre*_, precontrast TWIST sequence; *TWIST*_*N*_, *N*th postcontrast sequence; *MIP*, maximum intensity projection; *T1-w*_*Pre*_, precontrast T1-weighted sequence

The proposed deep learning system had three main stages: breast region segmentation, MIP generation, and abnormality prediction (Fig. 3).

For breast segmentation, a previously reported 3D U-Net [18] was used to generate the mask of the breast region. The segmentation was performed on a T1-weighted fat-suppressed sequence acquired before contrast agent injection. The obtained masks were then mapped onto TWIST sequences by shape resizing and FOV (field of view) alignment. Then, the breast area was divided into left and right segments from the middle of the mask.

At the stage of MIP generation, only the last four TWIST acquisitions out of the fourteen postcontrast phases were used. Previous research shows that the time of arrival of benign lesions may be much longer than that of malignant lesions [19, 20]; thus, most of the early MIPs contained no enhancing lesions. Therefore, to identify as many lesions as possible and reduce computational burden, in this study, the generated MIP images were then used to train the deep learning model.

A ResNet-34 model [21], which was pretrained on the ImageNet dataset, was modified and retrained for abnormality prediction. The output of the last fully connected layer of the model was changed to 2 to fit the task. The training data were then used for transfer learning, and validation data were used for hyperparameter tuning. The tasks used for training were the presence or absence of visible lesions in the MIP image. During the training process, image augmentation was applied with random horizontal flipping, random rotation within 10°, and random scaling within 10%. The batch size was set to 4, and the Adam optimizer was used. The final model was obtained by 60 epochs of training with an initial learning rate of 10^−4^. During inference, each of the 4 MIP images from a single breast was input into the deep learning model; if any of these images was predicted to be positive, the breast was then categorized as abnormal. The breast was only categorized as lesion free when all 4 MIP images were predicted to be negative.

### Model calibration and evaluation

To leverage the trained model to identify as many abnormal MRI exams as possible, a probability threshold that ensures a lower false negative rate (FNR) is preferable. On the other hand, the effect of the false-positive rate (FPR) on the workload in the screening workflow should also be considered. To illustrate the relationship between FNR and FPR, the detection error trade-off (DET) curve for the validation set was generated. Thresholds that corresponded to a sensitivity of 100% or 95% and a negative predictive value (NPV) above 98% on the validation set were then selected as high sensitivity thresholds.

To evaluate the prediction performance of the proposed deep learning system, receiver operating characteristic (ROC) curves on the independent test set were generated and the area under the receiver operating curve (AUC) was calculated. Sensitivity, specificity, positive predictive value (PPV), and NPV were also calculated for the default and high sensitivity thresholds, respectively. Furthermore, to help explain the decision-making of the classification model, Gradient-weighted Class Activation Mapping (Grad-CAM) was used to produce a coarse localization map, highlighting class-discriminative regions in each MIP image.

Strong background parenchymal enhancement (BPE) has been reported to be associated with higher abnormal interpretation rates and lead to higher rates of unnecessary biopsies [22]. The percentage of each category of BPE in false positive and false negative predictions was examined to illustrate the effect of BPE on the model output.

To evaluate the effect of the deep learning system on the clinical workflow, we simulated the scenario in which negative results from the TWIST sequences did not require patients to undergo further work-up or require radiologists to interpret those examinations. The reduced acquisition time and percentage of excluded MRI examinations were calculated based on this scenario.

### Statistical analysis

Medcalc (version 19.6.1 Medcalc Software Ltd) and scikit-learn (version 0.24.1; https://scikit-learn.org) were used for statistical analyses. The 95% confidence intervals (CI) for the AUCs were computed with DeLong’s method [23], 95% Clopper-Pearson CI for sensitivity and specificity, and 95% standard logit CI [24] for PPV and NPV were also reported.

## Results

### Patients and lesions

The training and validation sets consisted of 339 patients (median age ± standard deviation, 44 ± 11 years; range, 22–80 years) who underwent 659 breast screening MRI examinations. Among these, 494 examinations were used for model training, and 165 were used for validation. The left and right breasts in each examination were classified separately, which resulted in the identification of 118 abnormal breasts (lesion size ± standard deviation, 17.9 ± 17.4 mm; range, 5.0–110.0 mm) and 1200 normal breasts. Eighty-four of the abnormal breasts contained benign lesions (lesion size ± standard deviation, 13.7 ± 12.8 mm; range, 5.0–81.0 mm), while the other 34 contained malignant lesions (lesion size ± standard deviation, 25.1 ± 19.8 mm; range, 6.0–110.0 mm).

The independent test set consisted of 149 patients (median age ± standard deviation, 44 ± 15 years; range, 24–76 years) who underwent 178 breast screening MRI examinations. Fifty-five breasts were classified as abnormal (lesion size ± standard deviation, 24.0 ± 19.8 mm; range, 5.0–76.0 mm), and 301 were classified as normal. Twenty-five of the 55 abnormal breasts contained benign lesions (lesion size ± standard deviation, 15.0 ± 16.1 mm; range, 5.0–75.0 mm), and 30 contained malignant lesions (lesion size ± standard deviation, 31.0 ± 19.4 mm; range, 6.0–76.0 mm). Detailed patient and lesion characteristics are provided in Tables 2 and 3.

**Table 2:** Patient and examinations characteristics

Characteristic	Training and validation	Independent test
No. of patients	339	149
No. of examinations	659	178
No. of single breasts	1318	356
Mean age of patients	44 ± 11	44 ± 15
BI-RADS assessment		
BI-RADS 1	161 (24.4)	34 (19.1)
BI-RADS 2	434 (65.9)	105 (59.0)
BI-RADS 3	27 (4.0)	6 (3.4)
BI-RADS 4	6 (1.0)	7 (3.9)
BI-RADS 5	4 (0.6)	2 (1.1)
BI-RADS 6	27 (4.0)	24 (13.5)
Magnetic field strength		
1.5 T	273 (41.4)	59 (33.1)
3.0 T	386 (58.6)	119 (66.9)
Background enhancement		
Minimal	263 (39.9)	66 (37.1)
Mild	204 (31.0)	55 (30.9)
Moderate	168 (25.5)	48 (27.0)
Marked	24 (3.6)	9 (5.0)
Fibroglandular tissue		
Almost entirely fat	106 (16.1)	32 (18.0)
Scattered	244 (37.0)	58 (32.6)
Heterogeneous	240 (36.4)	67 (37.6)
Extreme	69 (10.5)	21 (11.8)
Gene mutation		
Yes	218 (64.3)	56 (37.6)
No	107 (31.6)	88 (59.0)
Possible	14 (4.1)	5 (3.4)

Data in parentheses are percentage.

*BI-RADS* Breast imaging-reporting and data system.

**Table 3:** Description of lesions in the abnormal breasts

Lesion type	Training and validation	Independent test
Benign lesions	84 (71.2)	25 (45.5)
Adenosis	21 (17.8)	3 (5.5)
Fibroadenoma	12 (10.2)	7 (12.7)
Other*	51 (43.2)	15 (27.2)
Malignant lesions	34 (28.8)	30 (54.5)
Invasive ductal carcinoma	26 (22.0)	25 (45.5)
Invasivelobular carcinoma	3 (2.5)	1 (01.8)
Ductal carcinoma in situ	2 (1.7)	2 (3.6)
Micropapillary carcinoma	1 (0.8)	1 (1.8)
Apocrine carcinoma	1 (0.8)	0
Mucinous carcinoma	1 (0.8)	1 (1.8)
Lesion size (mm) †		
Overall	17.9 ± 17.4 (5.0–110.0)	24.0 ± 19.8 (5.0–76.0)
Malignant	25.1 ± 19.8 (6.0–110.0)	31.1 ± 19.4 (6.0–76.0)
Benign	13.7 ± 12.8 (5.0–81.0)	15.0 ± 16.1 (5.0–75.0)

Unless otherwise indicated, data in parentheses are percentage.

*The “Other” category included enhancement around fat necrosis, scar tissue, hyperplasia, atheroma cyst, regional background enhancement, and other benign-appearing enhancement not specified.

†Data are ± standard deviation; data in parentheses are range of size.

### Model calibration

**Fig. 4:**
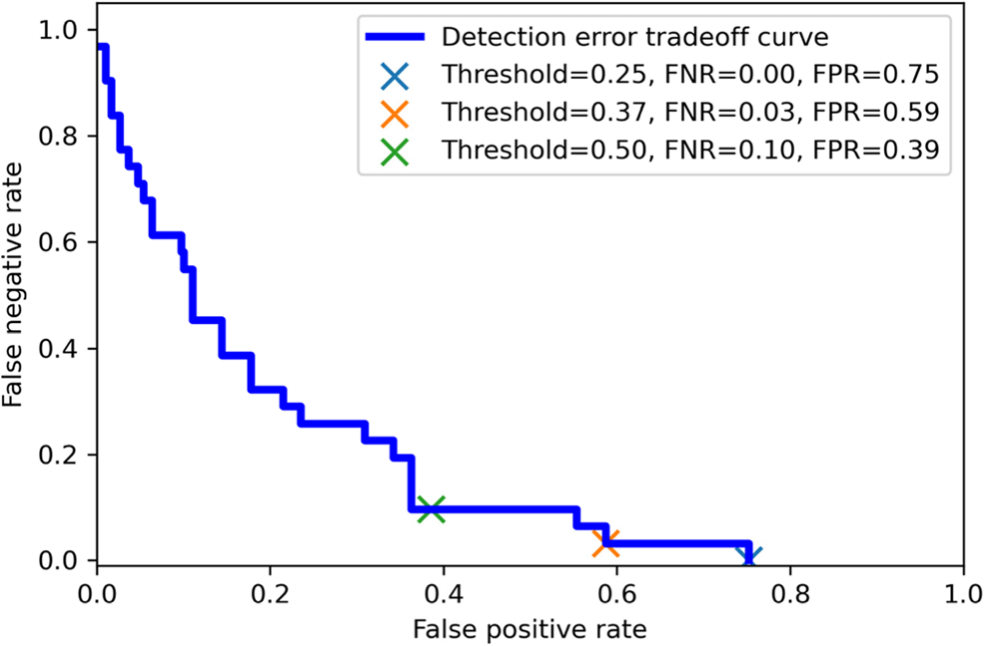
Detection error trade-off curve on the validation set. *FPR*, false-positive rate; *FNR*, false negative rate; *DET*, detection error trade-off

The DET curve on the validation set, which illustrates the trade-off between FPR and FNR with the threshold ranging from 0 to 1, is shown in Fig. 4. Two cutoff thresholds were selected based on the DET curve. With a threshold of 0.37, a sensitivity of 97% (30 of 31, 95% CI: 83%; 100%) and NPV of 98% (123 of 124, 95% CI: 95%; 99%) were achieved. With this threshold, one breast with a benign lesion (chronic active inflammation with fat necrosis, 38 mm) was misclassified in the validation set, and no malignant lesions were missed. With a threshold of 0.25, a sensitivity of 100% (31 of 31, 95% CI: 89; 100) and NPV of 100% (74 of 74) were achieved with no lesion missed.

### Independent test

**Fig. 5:**
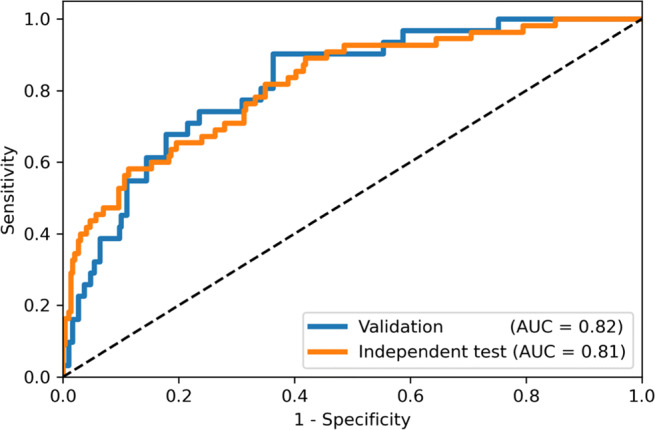
Receiver operating characteristic curves on the validation and independent test sets. The area under the receiver operator characteristic curve on the validation set and independent test set was 0.82 (95% confidence interval: 0.74; 0.88) and 0.81 (95% confidence interval: 0.75; 0.88), respectively

On the independent test set, the model achieved an AUC of 0.81 (95% CI: 0.75; 0.88) (Fig. 5). With the threshold of 0.37, a sensitivity of 95% (52 of 55, 95% CI: 85%; 99%) and NPV of 97% (106 of 109, 95% CI: 92%; 99%) were achieved, while with the threshold of 0.25, a sensitivity of 98% (54 of 55, 95% CI: 90%; 100%) and NPV of 98% (55 of 56, 95% CI: 89%; 100%) were achieved. The classification performance with each threshold is summarized in Table 4.

**Table 4:** Performance of the model on validation and independent test set for different threshold setting

	Validation				Independent test			
Threshold	Sensitivity (%)	Specificity (%)	PPV (%)	NPV (%)	Sensitivity (%)	Specificity (%)	PPV (%)	NPV (%)
0.25	100	25	12	100	98	18	18	98
	(31/31)	(74/298)	(31/255)	(74/74)	(54/55)	(55/301)	(54/300)	(55/56)
	[89, 100]	[20, 30]	[11, 13]	N/A	[90, 100]	[14, 23]	[17, 19]	[89, 100]
0.37	97	41	15	98	95	35	21	97
	(30/31)	(123/298)	(30/205)	(123/124)	(52/55)	106/301)	(52/247)	(106/109)
	[83, 100]	[36, 47]	[13, 16]	[95,100]	[85, 99]	[30, 40]	[19, 23]	[92, 99]
0.50	90	61	20	98	91	52	26	97
	(28/31)	(183/298)	(28/143)	(183 / 186)	(50/55)	(158/301)	(50/193)	(158/163)
	[74, 98]	[56, 67]	[15, 21]	[95, 99]	[80, 97]	[47, 58]	[23, 29]	[93, 99]

Numbers in parentheses are the numbers of single breasts. Numbers in brackets are 95% confidence intervals.

*PPV* positive predictive value, *NPV* negative predictive value, *N/A* not available

**Fig. 6:**
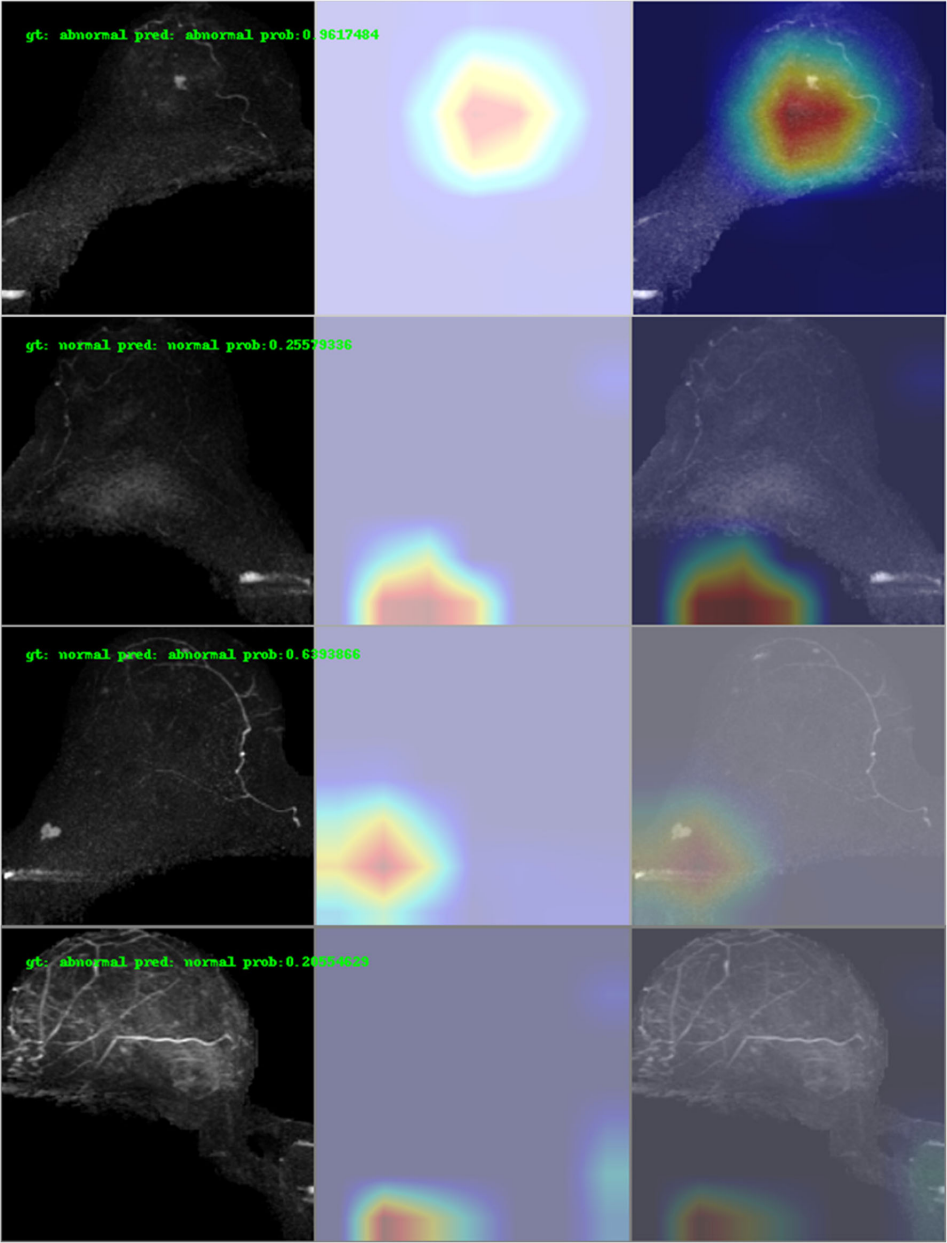
True positive, true negative, false positive, and false negative examples from the independent test set and corresponding heatmaps generated with gradient-weighted class activation mapping (Grad-CAM)

Heatmaps generated with Grad-CAM indicate that, for positive predictions, the model made the decision mainly based on the enhanced regions in the breast parenchyma, while for negative predictions, the model’s focus was outside of the breast parenchyma. Examples are shown in Fig. 6.

The percentage of each BPE level in the false predictions of the independent test set was also investigated. For false negative predictions, 1 had minimal BPE and 2 had moderate BPE; meanwhile for false positive predictions, 35.9% were minimal BPE, 30.7 % were mild BPE, 25.6% were moderate BPE, and 5.1% were marked BPE.

### Standard workflow vs. triage

When applying a threshold of 0.37 on the independent test set, 3 breast lesions were misclassified by the model; one contained a malignant lesion (mucinous carcinoma, 8 mm, BI-RADS 6), while the other two contained benign lesions (one with fibroadenoma, 9 mm, BI-RADS 4 and one not biopsied, 6 mm, BI-RADS 2). With the threshold of 0.25, only the one with fibroadenoma was misclassified as normal; no breasts with malignant lesions were missed.

Despite the possible risk of misclassifying breast lesions, with a threshold of 0.37, 109 breasts were triaged as normal and 247 as abnormal, resulting in a workload reduction of 30.6% (109 of 356) at the breast level or 15.7% (28 of 178) at the examination level. If the threshold was further lowered to 0.25, 56 breasts were triaged as normal, while 300 were triaged as abnormal, resulting in a workload reduction of 15.7% (56 of 356) at the breast level and 6.2% (11 of 178) at the examination level. Furthermore, 30.2% (982.2 of 3253.8 min) or 16.6% (538.8 of 3253.8 min) of scanner time could be saved over 178 examinations under different settings if scanning was only continued when an abnormality was detected by ultrafast MRI.

## Discussion

In this study, we combined clinical experience with artificial intelligence for the purpose of improving the efficiency and accessibility of breast MRI screening. A deep learning model was developed to identify normal ultrafast breast MRI examinations.

The model achieved an AUC of 0.81 (95% CI: 0.75; 0.88) on an independent test set. High sensitivity (95% and 98%) and negative predicted values (97% and 98%) were obtained by applying different thresholds (0.37 and 0.25). When integrated into the workflow, the model has the potential to reduce radiologists’ workload by excluding normal scans and improving throughput by reducing scanning time. Moreover, the heatmap generated with Grad-CAM could also support radiologists’ image interpretation by identifying possible lesions in the MIP image.

Although a conservative strategy was adopted, there were still false negative predictions. All the missed lesions were smaller than 10 mm, and the relatively small size may be the main reason that the deep learning model did not detect them. One malignant lesion (a mucinous carcinoma) was missed when using the threshold of 0.37. However, it should be noted that there was only one mucinous carcinoma in the training dataset, and the scarcity of this rare cancer might have caused the model to be insufficiently trained to identify it. For false positive predictions, the percentages of minimal, mild, moderate, and marked BPE were 35.9%, 30.7%, 25.6%m and 5.1%, respectively. Compared with the BPE distribution in Table 2 (37.1% minimal, 30.9% mild, 27.0% moderated, and 5% marked), it is hard to make a conclusion that BPE had a negative impact on the classification of MIPs in TWIST. Meanwhile, 134 of the 195 false positive prediction were BI-RADS 2, and 113 were assessed within heterogeneous and extreme FGT. This finding indicates that proper handling of dense and BI-RADS 2 breasts may be the key to reducing false positives in the future.

Similar models have been developed or evaluated in other studies on screening [25, 26]. Verburg et al [27] developed a classification model with 4581 MRI examinations of extremely dense breasts; the model could help exclude 39.7% of the MRI examinations without lesions and preserve 90.7% with lesions for radiologic review. Rodriguez-Ruiz et al [28] and Yala et al [9] showed that AI could help reduce mammogram screening workload by 17% or 19.3% with a sensitivity of 90.6% or 90.1%, respectively. Raya-Povedano et al [29] also reported a 29.7% workload reduction for tomosynthesis screening with a sensitivity of 84.1%. Even though the modality is different, the challenge of using AI in triaging is the same: a lower threshold is safer but less efficient, and the trade-off between the risk of missing breast cancer and the reduction of workload makes the threshold difficult to determine.

One of the limitations in our study is that the model was developed with a high-risk population dataset collected from a single institution. This may affect the generalizability of this study. External validation with diverse populations is necessary before clinical implementation. Another limitation of this study is that the cancer rates in the independent test set and the training and validation sets were not equal. These two subsets of data were downloaded separately from the same picture archiving and communication system via a time-consuming acquisition process. This ensured independence but may have introduced discrepancies in the reported results. In addition, this study was limited in exploring the real effect of the deep learning model in the triage workflow. A double-blind, randomized clinical trial may be necessary to further evaluate the performance of the model. Moreover, the proposed method used the 3D mask derived from T1-weighted fat-suppressed sequences, which may introduce systematic error. Developing a TWIST-based segmentation method might help further improve its performance. Furthermore, the MIP images used in this study are only generated in the axial plane, and potential masking effects may hinder the deep learning model from achieving better performance. Evaluation of multiplanar MIPs may be a potential solution to address MIP masking effects.

In conclusion, the classification of ultrafast breast MRI examinations with a deep learning model in the workflow may be a promising method to improve the efficiency and accessibility of breast MRI screening. Reduced scanning and interpretation time could result in significantly lower breast MRI screening costs, making it possible to provide MRI screening for a wider population.

## Supplementary Information

ESM 1 (DOCX 3222 kb)

## Abbreviations

AI: Artificial intelligence
DCE-MRI: Dynamic contrast-enhanced MRI
DET: Detection error trade-off
Grad-CAM: Gradient-weighted class activation mapping
MIP: Maximum intensity projections
NPV: Negative predictive value
PPV: Positive predictive value
TWIST: Time-resolved angiography with stochastic trajectories

## Declarations

### Guarantor

The scientific guarantor of this publication is Assoc. Prof. Peter M. A. van Ooijen.

### Statistics and biometry

No complex statistical methods were necessary for this paper.

### Methodology

• Retrospective

• Case-control study

• Performed at one institution
