# Supplementary material for: Using deep learning to safely exclude lesions with only ultrafast breast MRI to shorten acquisition and reading time
Source: Eur Radiol. 2022 May 26;32(12):8706–15. doi: 10.1007/s00330-022-08863-8 (PMC9705471; doi:10.1007/s00330-022-08863-8)
Supplement: Supplementary file 1 — (DOCX 3222 kb) [file 330_2022_8863_MOESM1_ESM.docx]

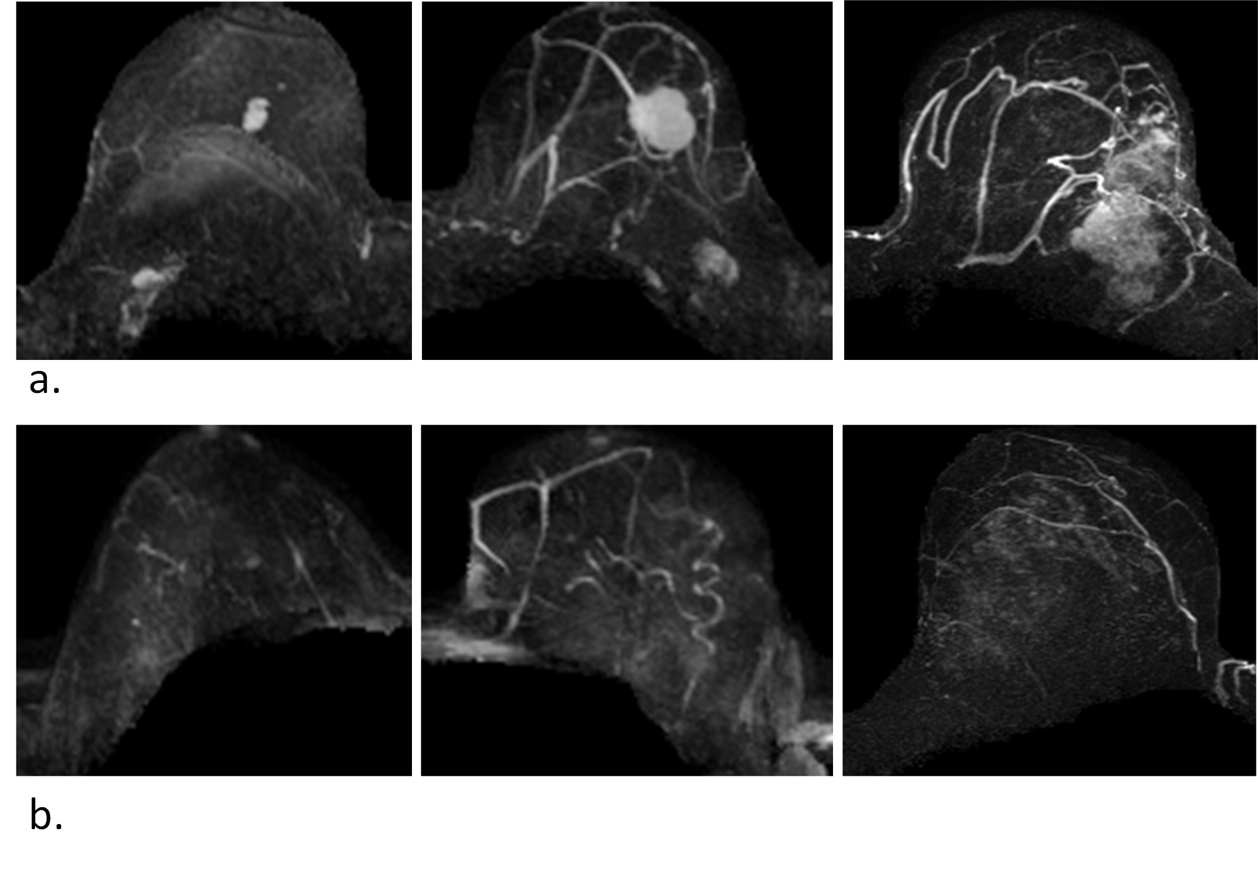


**Figure S1**: Example maximum intensity projection (MIP) of the time-resolved angiography with stochastic trajectories (TWIST) sequences of labeled breasts. (**a**) Abnormal and (**b**) Normal Samples.
